# Effects of Ozaki procedure on hematological and biochemical parameters

**DOI:** 10.1590/1806-9282.20240944

**Published:** 2024-12-02

**Authors:** Yasin Ozden, Ferdi Peynirci, Seyma Ozden, Mutlu Senocak, Yavuz Sensoz, Ilyas Kayacioglu

**Affiliations:** 1 Dr Siyami Ersek Thoracic and Cardiovascular Surgery Training and Research Hospital, Department of Cardiovascular Surgery – İstanbul, Turkey.; 2 Sureyyapasa Chest Diseases and Thoracic Surgery Training and Research Hospital, Immunology and Allergy Clinic, Department of Chest Diseases – İstanbul, Turkey.

**Keywords:** Heart surgery, Aortic valve, Aortic valve stenosis, Aortic valve insufficiency

## Abstract

**INTRODUCTION:**

The Ozaki procedure is an open-heart surgery technique used in aortic valve surgery. We investigated the effect of the Ozaki procedure on postoperative hematological and biochemical parameters as in other open-heart surgery applications.

**METHODS:**

A total of 42 patients between December 2020 and December 2022 who underwent the Ozaki procedure were analyzed retrospectively. Patients without any comorbid cardiac pathology were included in the study. Preoperative 3rd day, postoperative 6th hour, postoperative 24th hour, postoperative 7th day, and postoperative 30th day hematological and biochemical parameters of 42 patients with screening results were examined.

**RESULTS:**

Postoperative values were compared according to preoperative values. According to this, hematocrit and hemoglobin values were significantly lower at all times postoperatively. While white blood cells were found to be significantly higher until the postoperative 7th day, platelets were found to be significantly lower. The highest levels of creatine, blood urea, nitrogen, and aspartate aminotransferase were detected at the postoperative 24th hour, while the highest level for alanine aminotransferase was observed at the postoperative 7th day.

**CONCLUSION:**

As with all open-heart surgery methods, the Ozaki procedure may cause some changes in hematological and biochemical parameters in the postoperative period. So, this situation is the same as in other open-heart surgery methods.

## INTRODUCTION

The Ozaki procedure is an aortic valve neocuspidization method that has become increasingly popular recently and can be used in the treatment of all aortic valvulopathies (aortic stenosis, aortic regurgitation, aortic valve endocarditis), regardless of whether the aortic valve is tricuspid, bicuspid, or unicuspid. It has better hemodynamics compared to the biological and mechanical valves. In addition, there is no need to use anticoagulants for life in mechanical valves^1^.

This method, which was first applied in 2007, has very good mid-term hemodynamic results and durability^2^. The Ozaki procedure has been demonstrated by many studies that hope short- and medium-term results are successful and long-term results will be good^3^.

We have started to apply this technique in our clinic in recent years in order to keep up to date with this technique, whose popularity is increasing in the world. As with any new procedure, we had a learning curve in this technique. Since we have started this procedure routinely in patients suitable for aortic valve pathologies and have reached a sufficient number of patients for the literature, we conducted a study that included changes in the postoperative (postop) hematological and biochemical parameters of the patients for whom we applied this procedure.

## METHODS

Patients who were operated on with the Ozaki procedure for isolated aortic valve pathology (without additional cardiac pathology) between December 2020 and December 2022 were retrospectively screened. Preoperative (preop) 3rd day, postop 6th hour, postop 24th hour, postop 7th day, and postop 30th day hematological and biochemical parameters of 42 patients with screening results were examined. Postoperative values were compared according to preoperative values.

### Statistical analysis

The statistical analyses of the study were conducted using the trial version of SPSS 22.0 (SPSS Inc., Chicago, IL). Quantitative variables were assessed for normal distribution using Kolmogorov–Smirnov test. For dependent measures that followed a normal distribution, paired t-tests were used, and for those that did not follow normal distribution, Wilcoxon tests were used. Descriptive statistics for normally distributed quantitative variables were presented as mean±standard deviation, while non-normally distributed quantitative variables were presented as interquartile range (IQR). Descriptive statistics for qualitative variables were expressed as frequency (%). A significance level of p<0.05 was considered statistically significant.

### Declarations

Study approval was obtained from the institution’s scientific committee (Approval no: E-28001928-604.01.01). This study was conducted in accordance with the Declaration of Helsinki. Individual consent for the study was waived because of the retrospective nature of the study and because the analysis used anonymous clinical data.

## RESULTS

In our study, 26 of the patients were female and 16 were male. The mean age of the patients was 65. Table 1 shows the preoperative demographic values of the patients. Preop and postop 6th hour, postop 24th hour, postop 7th day, and postop 30th day hematological and biochemical values of the patients were compared (Table 2). According to this, hemoglobin (HGB) and hematocrit (HCT) values, which show red blood cells from hematological parameters, were found to be significantly lower in the early and late postoperative period compared to the preoperative situation. When evaluated in terms of white blood cells (WBC), a significant increase was found on the 6th and, 24th hours and 7th days postoperatively, but there was no significant increase in the postop 30th day. This increase is characterized by an increase in the percentages of neutrophils (NEU %) in the WBC. On the contrary, significant decreases were observed in the percentage of lymphocytes (LYM %) during the same periods. Changes in the percentage of neutrophils and lymphocytes on the postop 30th day were not found significant here either. There is a different situation in thrombocyte (PLT) values, which is another hematological parameter. Accordingly, while a significant decrease was detected in the first 3rd postoperative period, a significant increase was observed in the last postop 30th day compared to the preop values.

**Table 1:** Preoperative demographic values of the patients.

Value	n=42
Age, years, mean±SD	65.52±11.57
Gender (female), n (%)	26 (61.9)
GFR, mL/min, mean±SD	79.30±17.55
DM, n (%)	19 (45.2)
HbA1c, %, mean±SD	6.16±0.75
Prior coronary angioplasty, n (%)	3 (7.1)
EF %. mean±SD	55.59±9.11
AS, n (%)	26 (61.9)
AR, n (%)	4 (9.5)
AS+AR, n (%)	12 (28.6)
Mean Gradient, mmHg, mean±SD	47.40±16.02
Max Gradient, mmHg, mean±SD	76.64±24.78
Vmax, m/s, mean±SD	4.27±0.81
PAP, mmHg, mean±SD	32.90±10.89

SD: standard deviation; GFR: glomerular filtration rate; DM: diabetes mellitus; HbA1c: hemoglobin A1c; EF: ejection fraction; AS: aortic stenosis; AR: aortic regurgitation; PAP: pulmonary artery pressure; Vmax: maximum velocity.

**Table 2:** Comparison of hematological and biochemical values of patients.

	Preoperative	Postoperative 6th hour	Postoperative 24th hour	Postoperative 7th day	Postoperative 30th day	p-value (postop 6th hour)	p-value (postop 24th hour)	p-value (postop 7th day)	p-value (postop 30th month)
HGB (gr/dL), mean±SD	12.66±1.95	9.81±1.03	8.89±0.91	9.34±1.42	11.13±1.20	**<0.001** *	**<0.001** *	**<0.001** *	**<0.001** *
HCT (%), mean±SD	38.46±5.22	29.76±3.01	27.02±2.61	29.03±4.29	34.10±4.63	**<0.001** *	**<0.001** *	**<0.001** *	**<0.001** *
WBC (n X 1000), mean±SD	8.01±2.17	15.46±5.92	13.83±3.93	11.31±3.74	8.55±2.76	**<0.001** *	**<0.001** **	**<0.001** *	0.143**
NEU (%), mean±SD	62.75±9.61	75.93±5.22	85.40±4.27	72.88±9.63	65.09±10.44	**<0.001** **	**<0.001** *	**<0.001** *	0.307*
LYM (%), mean±SD	26.94±8.46	10.94±4.89	7.86±3.06	17.35±7.66	24.85±9.20	**<0.001** **	**<0.001** **	**<0.001** *	0.284*
PLT (n X 1000), mean±SD	251.21±86.60	169.69±61.27	133.21±44.83	240.61±141.48	325.10±120.32	**<0.001** **	**<0.001** **	**0.020** **	**0.001** **
CRE (mg/dL), mean±SD	0.88±0.20	0.86±0.22	0.94±0.27	0.85±0.26	0.82±0.21	**0.035** **	**0.011** *	0.164**	0.081*
BUN (mg/dL), mean±SD	37.01±14.67	41.27±12.92	43.06±13.97	43.44±21.82	38.14±20.70	**0.029** *	**0.007** *	0.112**	0.818**
AST (IU/L), Median (IQR)	17.00 (8)	48.00 (23)	55.00 (48)	25.50 (20)	18.00 (10)	**<0.001** **	**<0.001** **	**<0.001** **	0.343**
ALT (IU/L), median (IQR)	15.00 (10)	16.00 (9)	20.00 (15)	23.50 (24)	14.00 (9)	0.464**	**0.031** **	**0.001** **	0.474**

*Paired sample t-test.

**Wilcoxon t-test. SD: standard deviation; HGB: hemoglobin; HCT: hematocrit; WBC: white blood cells; NEU: neutrophils; LYM: lymphocytes; PLT: thrombocyte; CRE: creatinine; BUN: blood urea nitrogen; AST: aspartate aminotransferase; IQR: interquartile range; ALT: alanine aminotransferase. Values that are statistically significant at p<0.05 are indicated in bold.

When we look at biochemical parameters, the situation is more variable. While an increase is observed in creatinine (CRE) values at the 6th and 24th hours postoperatively, this situation is not observed on the 7th and 30th postoperative days. While blood urea nitrogen (BUN) was found to be significantly higher in the postoperative 6th and postoperative 24th hours, no significant increase was observed in the later periods.

Aspartate aminotransferase (AST) showed a significant increase in the first three postoperative periods, this increase was not found at the postoperative 30th day. Finally, the increase in alanine aminotransferase (ALT) at the postop 6th hour and postop 30th day was not significant, but the increase in the postop 24th hour and postop 7th day was significant.

## DISCUSSION

There may be many complications that can occur after cardiac surgery. Among them, hemodynamic, circulatory, respiratory, renal, gastrointestinal, and hematological complications are the main ones^4^. Many postoperative parameters are monitored for the follow-up and treatment of these conditions. We discussed the hematological and biochemical parameter changes that may occur after the Ozaki procedure.

Cardiac surgery with cardiopulmonary bypass (CPB) can result in significant decreases in hematocrit. The HCT parameter is often used as an indicator of anemia in cardiac surgery^5^. Anemia is associated with many adverse outcomes that may occur in the postoperative period^6^. HCT reduction is caused by CPB (prime solution and red blood cell destruction), cardiac surgical procedure, intravenous fluids, and coagulopathy^7^. In addition, aortic stenosis and mechanical aortic valve replacement are serious causes of anemia and thrombocytopenia due to mechanical destruction^8^. Major cardiac surgeries are a challenge for the hemopoietic system. Significant changes are seen in the three main cellular components of the hemopolitical system. The causes of this situation are CPB, operative and postoperative bleeding, frequent pre- and postoperative blood tests, hemodilution due to prime volume and significant shift in intravascular volume, mechanical trauma of blood cells, therapeutic hypothermia, comorbidities, use of anticoagulant and antiplatelet drugs, and transfusion of blood products^9^. In studies in the literature regarding the change of hematological parameters after cardiac surgery, it has been observed that all three main hematological parameters undergo significant changes in the early period and gradually return to normal in the following periods. This process was determined to be 2–6 months on average^10^. Lako et al.^11^, in a prospective study involving 164 patients who underwent postoperative coronary artery bypass grafting (CABG), showed that erythrocyte, HCT, and HGB values reached the lowest values on the postoperative 3rd day compared to the preoperative values, and WBC and NEU rapidly reached their highest values on the 2nd postoperative day and decreased on the 1st postoperative day for lymphocytes. On the contrary, the lowest value was measured on the 2nd postoperative day for PLT. In the same study, the time for all these values to return to normal values was 1 month for HCT, HGB, WBC, NEU, and LYM. For PLT, it was shown that it reached the highest value on the 14th day and decreased to normal levels on the 21st day^11^. In light of these data, we obtained almost the same data results for the Ozaki procedure, which is a cardiac surgery method. Although a decrease in HCT, HGB, and PLT values was observed in our patients in the early postoperative period, this decrease regressed in the first postoperative month as a result of nutrition, recovery of the body, and adaptation of the valve.

The blood products we gave to the patients also played a role in the change in hematological parameters. For our patients, we routinely gave two fresh frozen plasmas after the operation, which might increase or decrease in HGB, HCT, and PLT values. In addition, erythrocyte suspension was given to 17 patients, whole blood replacement was performed in five patients, and platelet replacement was not performed in any patient.

Since WBC and NEU % are acute phase reactants, they increase in the early period and return to normal in the late period^12^.

Acute renal failure (ARF) is a common and serious complication after open-heart surgery and can be seen in up to 30% of patients after cardiac surgery. Even a slight increase in postoperative serum CRE and BUN can affect mortality, morbidity, hospital cost, and length of stay^13^. ARF is directly related to the processes of progression to chronic renal failure and even to dialysis dependence, which seriously affect the prognosis in post-operative cardiac surgery patients^14^. Cardiac surgery-associated acute kidney injury (CS-AKI) is seen in 30–50% of patients who have undergone surgery, especially for congenital heart disease^15^. In the studies, CS-AKI patients were generally found to be older and more common in the female population. Preoperative hypertension, diabetes, congestive heart failure, coronary artery disease; preoperative use of angiotensin-converting enzyme inhibitors and angiotensin receptor blockers, calcium ion channel blockers, and beta-blockers; and preoperative low baseline estimated glomerular filtration rate (eGFR), preoperative low hemoglobin level may present with CRE elevation even if there is no CS-AKI at postoperative discharge^16^. Many recent studies have been developed for the diagnosis of AKI, but CRE and BUN still form the cornerstone of diagnosis in daily clinical practice^17^. We found significant increases in CRE and BUN values at the end of the first 24 h of the acute period. An unexpected decrease in the CRE values was observed within the 6th hour after the operation. We thought that this might be due to the increased plasma volume due to intravenous fluids given during and immediately after the operation. This significant residual observed in the first periods returned to normal values as of the 1st week. The Kidney Disease: Improving Global Outcomes (KDIGO) criteria separates CS-AKI into three stages. Stage 1 is an increase of ≥0.3 mg/dL in CRE levels within 48 h, which can be seen in approximately 40% of patients. Stage 1 regresses after 1 week with a decrease in CRE values in cases where no additional pathology develops^18^. This change in CRE values correlates with CRE values in patients who have undergone the Ozaki procedure in our study.

AST is a nonspecific cardiac marker^19^. In a study of 13,505 patients examining the effect of AST values on survival after CABG, they found that AST was an independent predictor of long-term all-cause mortality^20^. A significant AST and ALT increase in the first three postoperative days in patients with CABG has been shown in studies. It has been shown to be caused by the CPB period and hypotension during this time^21^. In Theodoraki’s study, AST and ALT values were found to be significantly higher on the postoperative 1st and 7th days after cardiac surgery, but this situation was evaluated as an acceptable clinical course^22^. In our study, we found that there was a significant increase in AST and ALT during the same periods. It was clinically acceptable for us as well, and in the later period, these values decreased.

## CONCLUSION

The Ozaki procedure is an open-heart surgery technique for aortic valve surgery. As with all open-heart surgery methods, it may cause some changes in hematological and biochemical parameters in the postoperative period. So, this situation is the same as in other open-heart surgery methods.
